# Kava-Kava in Gonorrhœa

**Published:** 1893-11

**Authors:** 


KAVA-KAVA IN GONORRHŒA.—Dr. John E. Bacon has studied the physiological action of the drug, which contains a crystaline principle, methysticin, and acrid resin, kavin. He has used a fluid and a solid extract of the root. A full dose of the fluid extract gives rise to a pronounced anæsthesia of the tongue and mucous membrane of the mouth and throat, lasting ordinarily about an hour. No irritation is produced by the fluid extract, but the solid, brought in contact with a mucous membrane, causes considerable pain, followed after a time by a complete loss of sensibility which persists for a period varying from twelve hours to three days. Therapeutic doses of the fluid extract (one-half to one drachm every four hours) increase the force and frequency of the pulse, increase the amount and diminish the specific gravity of the urine, and render it alkaline. He reports eighty-two consecutive cases of gonorrhœa. It relieves the *ardor urinæ*, scalding, and painful micturition, and by its anæsthetic effect lessens the pain and irritation. It will control and cure an ordinary case in from fifteen to thirty days without recourse to any topical treatment. He believes that it does this by controlling the symptoms, and by a certain antiseptic action due to tts intense alkalinity. Stricture very rarely follows an attack. In five cases only did failure occur, and in these the urine remained acid in spite of large doses of the remedy.—*American Therapist*, 1893, No. 12, p. 304.

[To those who have cofidence in the internal treatment of gonorrhœa we commend this paper. This remedy has enjoyed an excellent reputation in France for many years, and our observations, in the main, confirm the conclusions presented.—R. W. W.]—*Amer. Jour. Med. Science.*

